# Memorial to Dr. W. Xavier Sudduth

**Published:** 1916-01

**Authors:** William P. Cooke, A. I. Hadley, Henry H. Piper


MEMORIAL TO DR. W. XAVIER SUDDUTH. Dr. W. Xavier Sudduth, a distinguished associate fellow of the American Academy of Dental Science, died at Roundup, Montana, May, 1915.

Dr. Sudduth graduated from Illinois Wesleyan University, 1873, receiving the degree of M. A., graduated from Philadelphia Dental College 1881, and also studied in Heidelberg and Vienna, 1888-1889. He practiced dentistry in Illinois for about two years, when he became connected with the University of Minnesota as Secretary of the College of Dentistry and Professor of Embryology, Oral Surgery and Pathology and was Dean from 1892-1895. He was also much interested in histology and the therapeutic use of hypnosis. His clinical work was oral surgery.

Dr. Sudduth was a man of marked ability, integrity, and resources. He wrote a great deal, contributing to text books, and at one time was Editor of the International Dental Journal. He went to Montana from Minnesota, taking up his special hobby of horticulture and cattle-raising. He was much interested in the subject of cattle-feeding which led him to the raising of alfalfa. He was known as the ''Alfalfa King.''

Resolved: That in the death of Dr. W. Xaxier Sudduth the Academy mourns one of its most distinguished associate fellows, a man of marked ability and one who was ever an honor to his profession..

William P. Cooke,
A. I. Hadley,
Henry H. Piper.

